# Polymorphism of the *RNF165* Gene in American Mink (*Neogale vison*) as a Potential Factor Responsible for Resistance to Infection with the Aleutian Mink Disease Virus

**DOI:** 10.3390/genes16121417

**Published:** 2025-11-28

**Authors:** Ilona Mazurkiewicz, Andrzej Jakubczak, Marek Kowalczyk

**Affiliations:** 1 Institute of Biological Basis of Animal Production, University of Life Sciences in Lublin, 20-950 Lublin, Poland; ilona.mazurkiewicz@up.lublin.pl; 2 Department of Quality Assessment and Processing of Animal Products, University of Life Sciences in Lublin, 20-950 Lublin, Poland; marek.kowalczyk@up.lublin.pl

**Keywords:** American mink, RNF165 gene, Aleutian mink disease, molecular polymorphism

## Abstract

**Background:** Advances in American mink (*Neogale vison*) genomics have identified candidate genes linked to disease resistance, including *RNF165*, which is involved in immune response and virus–host interactions. Objectives: This study aimed to characterize genetic variation within the *RNF165* (Ring Finger Protein 165) gene in farmed and wild mink to identify polymorphisms potentially associated with resistance to Aleutian mink disease virus (AMDV). **Methods:** Two groups of mink were analyzed: farmed animals from Latvia (*n* = 50) and wild animals (*n* = 20) from Poland. The presence of viral DNA was verified by PCR amplification targeting the *VP2* and *NS1* viral protein genes. To assess polymorphism within *RNF165*, primers spanning eight exons were designed, optimized, and applied in PCR amplification. PCR products were sequenced and subjected to bioinformatic analysis. **Results:** Two single nucleotide polymorphisms were identified: a synonymous substitution at c.G141A and a missense mutation c.G596A (p.R199K). Both variants were present in farmed and wild populations; however, the G allele at position c.141 was nearly twice as frequent in farmed mink compared to wild mink (*p* < 0.05). In silico functional prediction (SNAP2) indicated that the p.R199K mutation may moderately affect RNF165 protein function. **Conclusions:** Owing to its high conservation and role in immune regulation, *RNF165* may serve as a promising candidate gene for molecular selection in breeding programs aimed at enhancing resistance to Aleutian mink disease (AMD).

## 1. Introduction

The first attempts to describe the mink genome were impeded by the lack of a reference genome, until Cai et al. [1] assembled a draft reference genome of the American mink using Illumina technology, generating 797 Gb reads. They identified 21,053 protein-coding sequences in the mink genome, and the structure of the reference genome was in agreement with the genetic map based on microsatellite sequences. Karimi et al. [2] provided information about the American mink genome at the chromosome level. Integration of the data led to the generation of an assembly containing 183 scaffolds 220 Mb in size. The American mink genome assembly (ASM_NN_V1) was 2.68 Gb in length, with 98.6% of the whole genome covered by 15 chromosomes. A total of 25,377 genes were predicted in the genome of American mink using the NCBI Eukaryotic Genome Annotation Pipeline. Genetic structures in farmed and wild mink have been analysed using data derived from various molecular markers, including microsatellite markers, mitochondrial DNA, and single nucleotide polymorphisms (SNPs). Microsatellite loci have been used to analyse the genetic structures of Wild American mink in Japan [3,4], Sweden [5] and Spain [6]. Information from mitochondrial DNA and 11 microsatellite loci was used to understand the genetic structure of American mink introduced to southern Chile [7]. Horecka [8,9] conducted a similar analysis of genetic variation in three mink populations, including farmed and feral populations in Poland and a wild population in North America. Genotypes obtained using 194 SNPs, generated by restriction site-associated DNA sequencing, were used to analyse the genetic structure of farmed and feral American mink populations in Poland and Denmark [10]. Karimi et al. [11] used Genotyping-by-Sequencing (GBS) sequencing to genotype American mink in Canada and obtained data containing 13,321 SNPs present on 46 scaffolds from 285 black mink. In another study, Karimi et al. [12] studied the genetic structure of American mink in Canada using whole genome sequencing (WGS) on 51 scaffolds from 100 farmed mink to obtain 100,000 SNPs.

The availability of a high-quality genome is not only essential to the development of genomic research on American mink, but would also be a valuable resource for comparative genomics and evolutionary studies of carnivorous mammals. American mink could be used as an animal model to study diseases common to humans and mink, such as deafness, Chédiak-Higashi syndrome, and susceptibility to coronavirus [2].

Karimi et al. [13] provided the first map of potential selection signals of the response to AMDV infection in American mink, as well as new information on genome regions controlling phenotypes of susceptibility to AMD. They discovered 63 genes associated with the immune response, liver cell proliferation, and reproductive traits. Using the hapFLK test (which differentiates a population on the basis of haplotypes) to detect molecular signatures of selection, they indicated two regions containing five candidate genes associated with the immune response and virus–host interaction, as well as reproduction, liver regeneration, and heart and kidney development, and thus with processes which may be disturbed due to replication of the virus. Given the above, the genes indicated by the authors may be of importance in terms of increasing resistance to infection and limiting its negative effects on the body.

The *RNF165* gene, together with four other genes (*SRSF5*, *SKOR2*, *SLC10A1* and *LOXHD1*), is one of the key genes described by Karimi et al. [13] as prognostic indicators of the immune response of mink to the AMD virus. They may be candidates for selection of mink that tolerate the AMD virus and may be involved in the body’s critical responses to AMDV infection. The detection of numerous loci subjected to selection for AMDV infection showed that genomic selection, i.e., selection of animals, may be a feasible approach to controlling this disease.

*RNF165* is a gene involved in critical responses to AMDV infection, such as the immune response and virus–host interactions. Through its activity as a ubiquitin ligase, the *RNF165* gene (RING Finger Protein 165), also called *ARK2C*, can cause the degradation of viral proteins [13]. The *RNF165* gene is involved in epigenetic programming of T cell phenotypes in the early stages of human development [14]. In mink, the *RNF165* gene is located in the region of the third chromosome encompassing positions from 150,694,340 to 150,759,578 base pairs (bp).

The aim of the study was to provide a molecular characterization of mink based on genetic variation with in the *RNF165* gene, which influences the development of the immune response, in a group of farmed and wild mink. It was hypothesized that some of the individuals tested would have molecular polymorphisms predisposing them to resistance to viral diseases. To verify the hypothesis, specific research objectives were formulated: to design primers flanking the *RNF165* gene; to identify polymorphic sites differentiating mink from other mustelid species on the basis of the reference sequence of the *RNF165* gene; and to determine the impact of individual substitutions on the functional effect of the coded protein. Therefore, an attempt was made to determine whether it is possible to conduct selection for resistant animals.

## 2. Materials and Methods

### 2.1. Tested Animals

All research procedures were approved by the Local Ethics Committee for Experiments on Animals of the University of Life Sciences in Lublin (decision no. 83/2009 and 55/2021).

Two experimental groups of animals were used in the study. The first comprised dead mink from a farm located in Latvia, and the other consisted of wild mink captured in Poland.

The biological material in the first experimental group comprised fragments of spleen tissue collected *post-mortem* from 50 individuals from the farm, which was chosen on the basis of specific criteria. The biological material was obtained from 20 females and 30 males, representing three colour variants: Brown, Silver and Pearl. The occurrence of the AMD virus in the herd had previously been confirmed by counterimmunoelectrophoresis—CIEP, which is routinely used in the diagnosis of AMDV [15]. Blood was collected by toenail clipping into capillary tubes, following local anaesthesia using 2% lignocaine. However, because the animals provided for the laboratory research were dead (having died at the age of 34 to 50 days), it was not possible to collect blood for serological testing of these individuals. The animals belonged to a herd in which CIEP confirmed the presence of AMD antibodies, and the minks died showing symptoms consistent with AMDV infection. For this reason, the presence of the virus was confirmed using a commercial qPCR test (Gensig AIDV advanced Kit—PrimerDesign™ Ltd., Eastleigh, UK).

The second group comprised 20 wild mink (6 females and 14 males) captured in Biebrza National Park in 2018 and 2019. All animals originated from Poland and exhibited a solid brown coat colour, visually resembling the standard Scanbrown phenotype.

### 2.2. DNA Extraction and AMDV Detection

DNA was isolated from tissues using the commercial DNeasy Blood and Tissue Kit (QIAGEN, Hilden, Germany), and the quality of the DNA was tested by electrophoresis in a 1% agarose gel using 1× TBE buffer, at a constant voltage of 80 V for 1 h. Samples were visualized using ethidium bromide (EtBr), which enables DNA intercalation, and observed in UV light. The gel image was archived using Scion Image software—version 4.0.2 (Scion Corporation, Frederick, MD, USA [16]).

Molecular diagnosis of AMDV was performed by polymerase chain reaction (PCR) using primers for fragments of genes encoding proteins VP2 (primers RP2, RP3—[17]) and NS1 (primer NS1—[18]) in the genome of the AMDV.

Both the farmed and wild mink underwent thorough PCR diagnostics, using three pairs of primers. Two of the pairs amplified two separate fragments of the gene encoding protein VP2, designated RP2 and RP3, while the third pair was used to amplify a fragment of the gene encoding protein NS1.

PCR was carried out using the AmpliTaq Gold 360 DNA Polymerase kit (Thermo Fisher Scientific Incorporated, Waltham, MA, USA). Each PCR used 3 μL of matrix DNA and 1 U of AmpliTaq Gold 360 DNA Polymerase (Thermo Fisher Scientific Incorporated, Waltham, MA, USA) in buffer supplied by the manufacturer. The PCRs were carried out in conditions in which the final MgCl_2_ concentration was 2.5 mM, the concentration of each dNTP was 0.8 mM, and that of each primer was 1.2 mM; the total volume of the reaction sample was 25 μL. The reaction took place under the following conditions: 95 °C for 10 min, 36 cycles of 95 °C for 30 s, 58 °C (RP2 and RP3) or 54 °C (NS1) for 45 s, 72 °C for 60 s, and 72 °C for 10 min in a Labcycler thermocycler (SensoQuest, Göttingen, Germany).

The presence and specificity of the PCR products was determined using electrophoresis on a 1% agarose gel at 80 V for 120 min. The gel was visualized and documented in UV light in the presence of ethidium bromide (EtBr) as a fluorescent tag, using Scion Image software—version 4.0.2.

### 2.3. PCR for RNF165 Gene

The RNF165 gene was selected on the basis of the results of Karimi et al. [13] who suggested that this gene may be involved in responses to AMDV infection. Next, sequences were sought in the Ensembl genomic information database using the name of the gene and species affiliation as criteria. In this way, files were obtained in FASTA format with highlighted exons, which were then used to design the primers.

PCR primers (×3 pairs) for eight exons of the *RNF165* gene were designed using the Primer Blast platform [19], under the following parameters: PCR product size—100–600 bp; primer melting temperature (T_m_) range—48–60 °C (maximum T_m_ difference—3 °C); GC content—40–60%; number of AT dinucleotide repeats—fewer than 4. When amplification was successful, only one pair was used, while in other cases amplification was carried out using two or three primer pairs. The sequences of the primers designed for individual exons of the *RNF165* gene are shown in Table 1. The amplification of each exon sequence was performed for all individuals.

To check the length of the resulting DNA fragments, the amplification products were subjected to electrophoretic separation on a 2% agarose gel, under the same conditions. Products of amplification were subjected to sequencing.

### 2.4. PCR Product Sequencing Reaction

PCR products were purified enzymatically with the EPPiC Fast Kit (A&A Biotechnology, Gdynia, Poland) to remove unbound primers and dNTPs. The reaction was carried out using the BigDyeTM Terminator v3.1 CycleSequencing Kit (Thermo Fisher Scientific Incorporated, Waltham, MA, USA) in the LabCycler thermal cycler (SensoQuest, Göttingen, Germany).

Asymmetrical PCR products were purified using the commercial ExTerminator kit (A&A Biotechnology, Gdansk, Poland). The sequencing PCR product, denatured and suspended in formamide, was subjected to capillary electrophoresis in the ABI Prism^®^ 3100 Avant Genetic Analyzer (Applied Biosystems, Foster City, CA, USA).

### 2.5. Bioinformatic Analysis

The sequencing data were analysed with Chromas 2.6.6 [20]. After accumulating repeats with the use of CAP3 software—version 3 [21], we ran them through NCBI Blast to check their degree of similarity. SNPs in these sequences were compared to the reference sequences. The reference sequence of the *RNF165* gene was obtained from the Ensembl database and aligned with reference sequences in the NCBI database using the Basic Local Alignment Search Tool (BLAST—version 2.17.0). Polymorphic sites were identified using MSA Viewer, built into the BLAST application, and the multiple sequence alignment (MSA) algorithm in Mega 11 software [22]. To illustrate the relationships between the American mink reference sequence and homologous sequences in other species and representatives of the group of farmed and wild mink, a phylogenetic tree was generated using the neighbour-joining (NJ) method. Amino acid sequences were compared in order to identify amino acid polymorphisms between the individuals analysed. The potential functional effects of the amino acid substitutions were determined using the SNAP2 tool [23], which is used to predict the effect of mutations on a protein’s functions.

## 3. Results

### 3.1. Sanger Sequencing

Following Sanger sequencing, chromatograms (in the form of AB1 files) were obtained for F and R primers for each of eight exons of the gene (Figures S1 and S2).

Contigs for each exon of the *RNF165* gene were compared with the reference sequence (XM_044244389.1) to determine repeatability, sequencing accuracy, and the presence of mutations. The fragments were combined into a complete gene sequence for each individual, which enabled identification of single nucleotide polymorphisms (SNPs), the type of indel, and analysis of their potential functional consequences.

### 3.2. Polymorphism of the RNF165 (ARK2C) Gene Sequence in the Family Mustelidae—Database Analysis

Polymorphism of the *RNF165 gene* sequence obtained from the Ensembl database with respect to other sequences deposited in the NCBI database was evaluated using the BLASTN algorithm. The ‘core_nt’ database was used for the analysis, with a query length of 1044 nucleotides. The results of the comparison are presented in Table 2. When the similarity threshold was increased to 98%, the only sequences remaining in the results were those of mammals belonging to the family *Mustelidae*, i.e., stoat (*Mustela erminea*), black-footed ferret (*Mustela nigripes*), ferret (*Mustela putorius furo*), European mink (*Mustela lutreola*), North American river otter (*Lontra canadensis*), Eurasian otter (*Lutra lutra*), European badger (*Meles meles*), and sea otter (*Enhydra lutris kenyoni*), as well as one member of the family *Phocidae*– grey seal (*Halichoerus grypus*). The greatest degree of homology was obtained for the predicted *RNF165* gene sequence in American mink, deposited under accession number XM_044244389.1.

**Figure 1:**
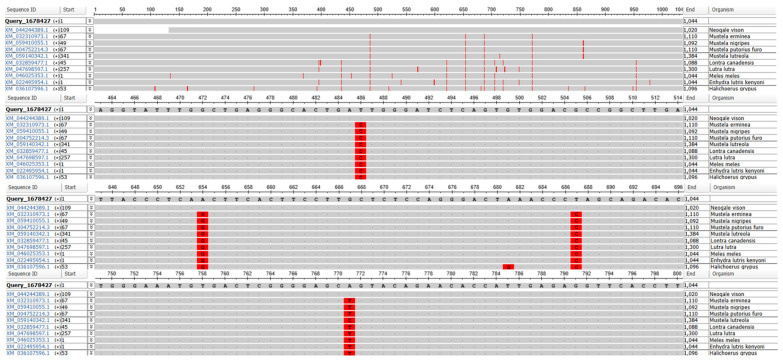
Polymorphic sites in the *RNF165* gene in relation to sequences of other species deposited in the NCBI database. Sequences included in analysis belong to the members of *Mustelidae* family and to the grey seal (*H. grypus*, family *Phocidae*). Red vertical lines indicate the polymorphic nucleotides between analyzed sequences.

Analysis with the MSA Viewer tool was used to determine molecular polymorphism of the RNF165 gene, whose sequence was downloaded from the Ensembl database, in relation to other sequences deposited in the NCBI database (Figure 1). In comparison with other species of the family Mustelidae, four polymorphic sites were identified: three Transition Mutations: T486C, A654G, T687C, and one transversion mutation: A771T/C.

### 3.3. Polymorphism of the RNF165 (ARK2C) Gene Sequence in the Tested Population

The BLAST was used to determine the percentage similarity of the RNF165 gene sequence in the tested mink (variants were designated _Ex 1–8, preceded by numbers from 1 to 6, with the numbers from 1 to 3 representing farmed mink and 4 to 6 representing wild mink). The point of reference in the analysis was the reference sequence from the Ensembl database. The results obtained in the sequence alignment process are presented in Table 3. The length of the sequences compared was 1044 nucleotides. The analysis revealed that the sequences obtained from the individuals had a very high degree of agreement with the reference sequence, ranging from 99.81 (for individuals 1_Ex.1–8, 2_Ex.1–8 and 4_Ex.1–8) to 99.90 (for individuals 3_Ex.1–8, 5_Ex.1–8 and 6_Ex.1–8). The sequence of individual 3_Ex.1–8 was clearly distinct from the average result for the group of farmed mink, while individual 4_Ex.1–8 was markedly different from the average for wild mink.

The BLAST was also used to determine polymorphic sites in the RNF165 gene sequence in the mink. The analysis identified two nucleotide polymorphisms: G141A transition (individuals 1_Ex.1–8, 2_Ex.1–8 and 4_Ex.1–8), G596A transition (observed in all individuals tested).

These mutations may have potential functional significance and require further analysis regarding their effects on the structure and function of the protein encoded by the *RNF165* gene.

**Figure 2:**
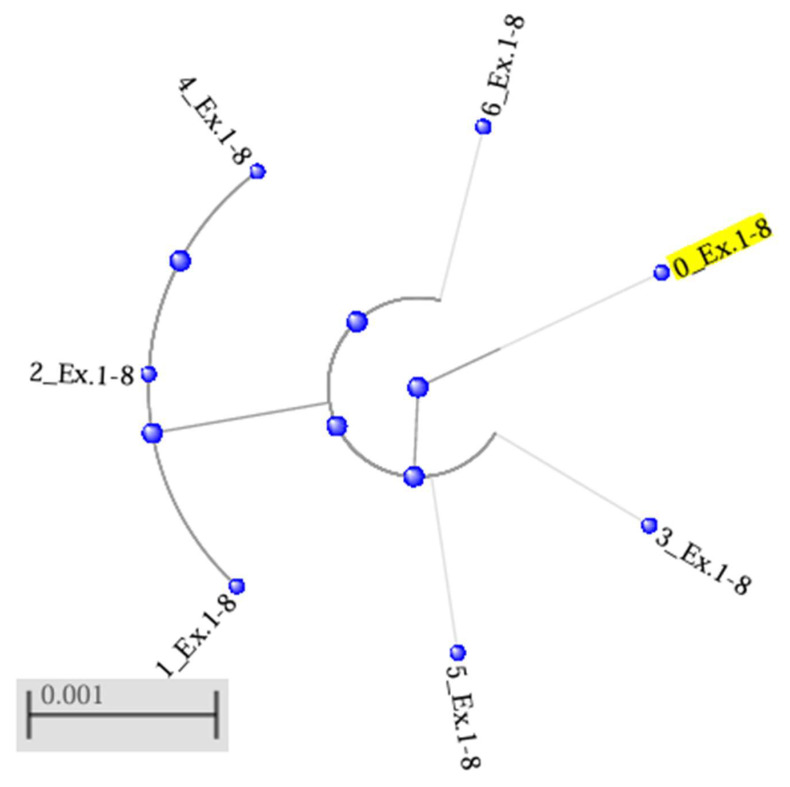
Phylogenetic tree for the *RNF165* gene, generated by the neighbour-joining (NJ) method using the BLAST, with a maximum acceptable sequence difference (Max Seq-Difference) of 0.75. The reference sequence was designated 0_Ex.1−8 (in yellow). Sequences of the *RNF165* gene belonging to the group of farmed mink are designated 1_Ex.1−8 to 3_Ex.1−8, while sequences of wild mink are designated 4_Ex.1−8 to 6_Ex.1−8.

The results of the phylogenetic analysis of the data on the RNF165 gene sequence were used to construct a tree by the neighbour-joining method, illustrating the genetic relationships between the variants occurring in the two populations (designated 1_Ex.1–8 to 6_Ex.1–8) and their phylogenetic distance from the reference sequence (0_Ex.1–8) (Figure 2). The resulting phylogenetic tree consists of two clades without branches. One contains the reference sequence, which occupies a peripheral location relative to the other three sequences in the clade, i.e., 3_Ex.1–8, 5_Ex.1–8 and 6_Ex.1–8. These sequences were located at a similar distance from the reference sequence, which indicates a lack of pronounced differences between them. The other, separate clade consists of the sequences of two farmed mink, 1_Ex.1–8 and 2_Ex.1–8, and one wild mink, 4_Ex.1–8. The results indicate a certain degree of genetic differentiation between sequences, with high similarity maintained within the main clade, which includes the reference sequence.

### 3.4. In Silico Analysis of the Impact of Polymorphism on the Functionality of the RNF165 Protein

Analysis of the amino acid similarity of the RNF165 protein showed a high degree of conservation. The sequences of the individuals (1_Ex.1–8 to 6_Ex.1–8) were 99.70% in agreement with the reference sequence (0_Ex.1–8), and identity between the same individuals was 100%, confirming the absence of structural differences in this protein between farmed and wild mink. Protein RNF165 (ring finger protein 165) is E3 ubiquitin-protein ligase ARK2C, and thus a protein performing enzymatic functions in eukaryotic cells. Amino acid sequences were aligned using the Mega 11 algorithm [21]. The alignment of the entire amino acid sequence of the protein RNF165 is presented in Figure S3. The sequence shows a high degree of conservation. An exception is position 199, at which an arginine (R) to lysine (K) substitution was noted in all individuals tested (both farmed and wild).

**Figure 3:**
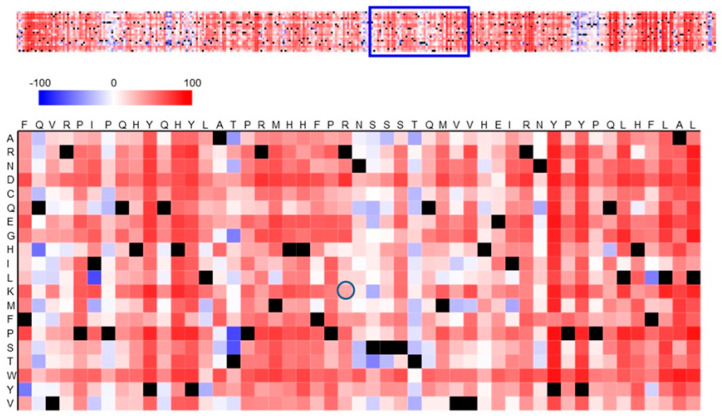
Prediction of the functional effects of the p.R199K polymorphism in RNF165. The amino acid substitution heatmap for the RNF165 protein was generated using the SNAP2 tool and illustrates the polymorphic amino acid in relation to the reference protein. Dark red indicates a high score (score > 50, strong signal for effect), white indicates weak signals (−50 < score < 50) and blue indicates a low score (score < −50, strong signal for neutral/no effect). Black designates the corresponding wild-type residues. The circle indicates a polymorphic amino acid—p.R199K.

Figure 3 shows a heatmap of the functional effects of the substitution in the protein RNF165. The polymorphism in the amino acid sequence involves arginine, which occurs in the reference sequence at position 199, while in all individuals tested it was replaced by lysine (at the position marked with a dark blue circle). According to the legend, the light red field indicates a weak effect of the p.R199K polymorphism of RNF165 on the functional effect. The value for this effect read on the heatmap is 33. In the context of the heatmap, this value indicates a moderate effect of the mutation on the function of ubiquitin-protein ligase ARK2C.

## 4. Discussion

The study on variation in the *RNF165* gene sequence in American mink, both farmed and wild, resulted in a number of important observations of functional, evolutionary, and immunological significance. This gene encodes RING finger-containing ubiquitin ligase, also called ‘Arkadia-like’, a protein with a proven role in many signalling pathways, including TGF-β and Wnt, and in neurogenesis processes [24,25]. Through its interaction with the Smad complex, this protein contributes to the degradation of repressors and modulation of the expression of target genes [24].

The *RNF165* sequences showed an extremely high degree of conservation within the population and between species within the family *Mustelidae*. Homology exceeding 99% indicates strong evolutionary pressure in the form of purifying selection, characteristic of genes encoding proteins of fundamental biological importance. This is consistent with previous reports by Zhang and Yang [26], indicating that conservation of the sequences of genes encoding proteins with important regulatory or developmental functions is often high among mammals. The authors found that the rate of evolution of proteins is inversely proportional to their functional importance—sequences of genes encoding key regulators change at a much slower rate [26].

### 4.1. RNF165 Gene Sequence Analysis

The *RNF165* gene is constructed of eight exons. Exons 1,3–4, and 6–8 of the *RNF165 gene* were determined to be conserved in the present study. The lack of nucleotide variation suggests that these regions are less susceptible to mutations and may perform key functions in preserving the structural and functional integrity of the RNF165 protein. Teraoka et al. demonstrated that even single changes in highly conserved exons may lead to serious biological disturbances, such as the substitution in the *ATM* gene leading to the E to K amino acid replacement [27]. This type of non-conservative substitution can therefore alter the structure or function of a protein, but will not necessarily inhibit its translation [27]. Similarly, the high degree of conservation of *RNF165* suggests that even subtle changes in the sequence may affect its functionality, despite its seemingly neutral biochemical nature.

Analysis of the results showed differences in the *RNF165* gene sequence obtained by Sanger sequencing, the NGS technique, and prediction using Gnomon bioinformatics algorithms. In contrast to Sanger sequencing and NGS, the Gnomon method involves drawing conclusions about the structure of a gene or transcript based on similarity to other known sequences, rather than on direct experimental confirmation in a laboratory [28]. The differences between the results obtained by NGS and prediction by the Gnomon method involved polymorphisms identified in the *RNF165* reference sequence (ENSNVIG00000000779) which were not consistent with the predictions for other *Mustelidae* species. Three transition mutations were noted, i.e., T486C, A654G and T687C, as well as a transversion: A771T/C. The *RNF165* reference sequence obtained by assembling scaffolds by the NGS method has guanine at position 141, while the Sanger method employed in the present study detected adenine in this position (*RNF165* G141A), which indicates a difference between the data obtained by these two methods. Another polymorphism confirming differences between the results of NGS sequencing and Sanger sequencing is the occurrence of guanine at position 596 in the reference sequence and adenine (G596A) in the sequence obtained in the present study by the Sanger method. The polymorphisms detected demonstrate the need for empirical validation of the data, especially in studies with potential application, such as in genetic selection in breeding.

### 4.2. Polymorphic Nucleotides in RNF165 Gene

Two single nucleotide polymorphisms (SNPs) were identified in the *RNF165* gene in the American mink populations: c.G141A and c.G596A. Both variants occur in both the farmed and wild mink populations, but the G allele at position c.141 has much higher allelic frequency in the farmed group—nearly twice as high as in the wild population (*p* < 0.05). This disproportion may indicate the potential impact of selection associated with domestication, especially if *RNF165* plays a role in physiological processes of importance for phenotypes selected in breeding (e.g., growth, adaptation to a closed environment, or neurological development). Polymorphism c.G596A is particularly interesting due to its presence in all of the individuals analysed. The same substitutions may reflect the evolutionary history of these populations, and their analysis may provide valuable information on adaptive and genetic processes taking place in mink populations. Further studies, including functional analysis of these polymorphisms, are required for a full understanding of their impact on expression of the gene and associated molecular mechanisms.

Michalska-Parda et al. [29] and Horecka [8,9] took up the problem of variation between farmed and wild mink using molecular marker information. Based on analysis of 11 microsatellite loci, Michalska-Parda et al. showed that farmed and free-living mink in Poland are two genetically close but separate groups [29]. Completely different results indicating the separation between farmed and wild populations, based on analysis of microsatellite polymorphism, differences in mtDNA, and selected nuclear gene sequences, were presented by Horecka [8] in a doctoral dissertation [8]. She demonstrated that the free-living Polish population was divided into two genetically distinct subpopulations: a north-eastern population with high genetic similarity to the indigenous mink population from North America, and a north-western population, with greater genetic similarity to farmed mink [8].

### 4.3. Effect of Detected Polymorphisms on the Amio Acid Sequence

We detected polymorphism p.R199K (Arg to Lys) which is considered conservative, because both are polar, basic amino acids of similar molecular volume. Although conservative amino acid replacements less often have adverse effects on the function of the protein, their biological significance cannot be ruled out; for example, they can affect protein–protein interactions, the stability of the protein’s structure, or cellular location [30]. In the case of RNF165, whose activity as a ubiquitin ligase is dependent on the precise structure of the RING domain, even seemingly minor changes can affect the protein’s functionality, which requires further functional studies.

The p.R199K mutation may lead to certain changes in the protein’s structure or function, but does not necessarily lead to a complete loss of its activity. The functional effect equal to 33 estimated by the SNAP2 tool suggests that the p.R199K mutation may have a moderate effect on the functioning of the protein RNF165, leading to partial impairment of its function, e.g., in the regulation of cellular signalling or in other processes associated with its functions. The effect of missense mutations is complex: on the one hand, they can be detrimental to host immunity. For instance, Nogales and DeDiego [31] analyzed polymorphisms in RIG-I and identified variants p.R71H (SNP rs72710678) and p.P885S (SNP rs138425677) that contributed to severe outcomes of influenza A infection. On the other hand, pathogens can act as selective forces. Enard et al. [32] suggested that viruses drive adaptive mutations in mammalian proteins, accounting for up to one-third of evolutionary changes in conserved regions of the human proteome.

Although the effect of the p.R199K mutation in the *RNF165* gene may seem mild in biochemical terms (due to the similar properties of arginine and lysine), the structural and functional context of position 199 is crucial. Bisimwa et al. [33] analysed sequence of *RelA* genes from ASFV-surviving and symptomatic pigs they identified 16 nonsynonymous SNPs, revealing two discrete RelA amino acid sequences—one in the surviving pigs, and the other in symptomatic pigs. Most of the polymorphic nucleotides were localized in exon 10 within the transactivation domain 2.

If R199 is located within or near the functional domain—RING, responsible for E3 activity—or at the site of contact with a partner protein, even such a conservative mutation may disturb the local spatial structure, alter the interaction surface, affect the specificity of the substrate, and even modify the degree of autoubiquitination of the protein. In the context of the functions performed by RNF165, the consequences of the mutation may include reduced catalytic efficiency, destabilization of the protein, and disturbances in the regulation of ubiquitination-dependent signalling pathways [34].

### 4.4. Possible Outome of Polymorphisms

The p.R199K substitution can influence the ability of the protein RNF165 to function as a ubiquitin ligase responsible for the attachment of ubiquitin chains to protein substrates, which initiates their degradation in proteasomes. According to Kelly et al., changes in the amino acid sequence can disrupt binding and reduce the enzymatic efficiency of ubiquitin ligase [35]. It can be concluded that if the p.R199K substitution affected a region involved in interactions with substrates, it could affect ubiquitination-dependent signalling processes, such as the response to oxidative stress, regulation of the cell cycle, or control of degradation of proteins regulating apoptosis. In addition to its enzymatic function as a transferase catalysing protein modification and its roles in signalling pathways and cell differentiation, RNF165 can also act as a virus receptor [34]. Therefore, the p.R199K polymorphism may modify the activity of RNF165 as a receptor for the AMDV. This mutation may also affect the stability of the protein RNF165 itself, because changes in structure can destabilize the protein and increase its susceptibility to degradation. If destabilization of the protein due to mutation causes it to degrade faster, this could have negative consequences for cellular functions, e.g., in the context of disturbances of protein homeostasis, which can be conducive to the development of neurodegenerative diseases or cancer. Kelly et al. report that the *RNF165* (*ARK2C*) gene is expressed specifically in the nervous system, and that its loss in mice causes defects in motor neurons which arise during development and lead to wasting and death before weaning [35].

Polymorphisms identified in the *RNF165* gene in mink may be associated with increased immunity or increased susceptibility to infection with the AMDV. Martino et al. [14] found that the *RNF165* gene encodes a protein involved in epigenetic programming of T cell phenotypes, which is significant in the immune response in humans. T cells are important immune cells responsible for recognizing and eliminating infected cells and pathogens, including viruses. Changes in epigenetic regulation of T cell phenotypes can influence the efficiency of the immune response. According to Martino et al. [14], the *RNF165* gene regulates the early stages of T cell programming, so changes detected in its sequence can affect the development of various subtypes of lymphocytes, and thus the body’s ability to effectively respond to viral infection. Markarian and Abrahamyan [36] report that once AMDV has entered the body, it undergoes constant and intensive replication. It exhibits particular tropism for lymphoid tissue, and the main target cells in adult mink are B and T lymphocytes. Aasted, in a study on the leukocyte system in AMD, showed a greater than two-fold increase in the number of T cells with the CD8+ phenotype, while the number of B and Th cells in the peripheral blood did not change significantly [37]. The continuous replication of the virus stimulates excessive secretion of specific antibodies by the immune system, as well as generalized plasmacytosis and monoclonal gammopathy [38]. Chen and Aasted [39] reported that hypergammaglobulinemia is observed in the serum of AMDV-infected mink, together with an increased antibody response to proteins NS1 and VP1/2 of the virus and a significant increase in the number of CD8+ lymphocytes in the blond. Similar changes were observed in the mesenteric lymph nodes and spleen. The authors noted that the thymus structure had vanished in severely infected mink, and CD3+ cells were found throughout the organ [39]. According to several authors, the size of the spleen, which performs important immune functions, has commonly been used to assess the immune response to various factors. In their study, the ratio of spleen weight to body weight was much higher in mink infected with AMDV [40,41,42]. Spleen enlargement was most likely caused by the presence of the pathogen, which means that the immune organs were in a state of high activation and more lymphocytes, mainly T cells, were produced [43]. Spleen enlargement in AMDV-infected mink is consistent with previous observations by Reichert and Kostro [38]. Persson et al. noted a difference in the relative weight of the spleen in male wild American mink, but found no differences in females [44].

The presence of the same *RNF165* polymorphisms in both groups of mink, irrespective of their health status, may suggest that the effect of these mutations on the clinical course of the disease is more complicated and depends on interactions with other genetic or environmental factors. *RNF165* mutations may be a marker associated with immunity, but the differences in immune responses between farmed and wild mink may be due to the joint action of genetic and environmental factors and the interactions between them. Further study is needed to fully understand how polymorphisms in the *RNF165* gene affect the immune response of mink. These mutations may influence various aspects of the immune response, the development of the disease, and clinical symptoms of infection.

## 5. Conclusions

The presence of both polymorphisms (c.G141A and c.G596AG) in the wild and farmed mink populations is indicative of the common origin of these genetic variants, but the varied frequency of alleles, i.e., the more frequent occurrence of the G allele at position 141 in the group of farmed mink, suggests the possible effect of selection pressure on adaptive traits promoting survival and reproduction in farm conditions. The p.R199K mutation can affect the resistance of mink to infection with the AMDV through epigenetic modulation of variation in lymphocyte subpopulations, including CD8+ lymphocytes, whose level undergoes changes during AMDV infection. Due to its role in immune regulation, the *RNF165* gene has characteristics of a candidate for a molecular selection marker in breeding programmes aimed at increasing resistance to AMDV. In particular, the p.R199K mutation should be further used as an indicator of susceptibility or resistance to infection.

In light of the above findings, the *RNF165* gene should be considered a key element of the molecular regulatory system in mink, potentially affecting fundamental developmental traits (neurogenesis) and immune mechanisms. Further study of the gene could be of significance for applications not only in breeding selection, but also in modelling of the immune response in mammals.

## Figures and Tables

**Table 1:** Characteristics of PCR primers for eight exons of the *RNF165* gene, with PCR product length [bp], melting temperatures [°C] percentage of GC pairs [%], experimentally determined optimal primer annealing temperatures (T_a_).

Exon	F/R *	Sequence	Product Length [bp]	T_m_ [°C]	GC [%]	T_a_ [°C]
1	F	GGCTATCTCGTGCTTCCAGT	265	53.8	55	59
	R	GGGACCCAAATTCGCCAAAC		53.8	55	
	F	GCTTCCAGTGTTTGGCTCTG	255	53.8	55	
	R	AGGGACCCAAATTCGCCAAA		51.8	50	
2	F	CTCCAGGTGCCCCTTTTCAA	451	53.8	55	59
	R	AGTAAAAGCTACCTCCCGCAC		54.4	52	
	F	CTCTCCAGGTGCCCCTTTTC	451	55.9	60	
	R	TAAAAGCTACCTCCCGCACAG		54.4	52	
	F	AAGGTCTCAGCATCCTCACG	433	53.8	55	
	R	CAGTAAAAGCTACCTCCCGCA		54.4	52	
3	F	TGTAAGCTGTAGGCTCAGGC	302	53.8	55	58
	R	GCCTCCTGCATCATTCAACA		51.8	50	
4	F	CTCTGAGTCTTGTGTCTGGGG	331	56.3	57	65
	R	GGAGGGGAGGACAGCATGAC		57.9	65	
5	F	ACTGACGAATCTCAGGGGTG	220	53.8	55	54
	R	GGGCGTACCTCATAGCTCTC		55.9	60	
6	F	CCCTCCCTCCGTATTGTGTG	203	55.9	60	58
	R	GGCTGACACACTAGCAGACT		53.8	55	
7	F	AGCTGCTCCAGGTAACGGAT	171	53.8	55	58
	R	ACAGAAGGAGCGGGTCTCTTA		54.4	52	
8	F	GTGATAGCCAGTATCGCGCC	277	55.9	60	58
	R	GTTTGGCTGCAAGGGCTATG		53.8	55	

* The number of primer pairs used for amplification depends on the effectiveness of amplification of the PCR product.

**Table 2:** Comparison of the *RNF165* gene sequence from the Ensembl database with other sequences available in the NCBI database.

Description	Max Score	Identity [%]	Length [bp]	Accession
PREDICTED: *Neovison vison* ring finger protein 165 (RNF165), mRNA	1685	100.00%	1609	XM_044244389.1
PREDICTED: *Mustela erminea* ring finger protein 165, mRNA	1906	99.62%	1738	XM_032310973.1
PREDICTED: *Mustela nigripes* ring finger protein 165, mRNA	1901	99.52%	1694	XM_059410055.1
PREDICTED: *Mustela putrius furo* ring finger protein 165, mRNA	1901	99.52%	1729	XM_004752214.3
PREDICTED: *Mustela lutreola* arkadia ring finger ubiquitin ligase 2C, mRNA	1895	99.43%	2024	XM_059140342.1
PREDICTED: *Lontra canadensis* ring finger protein 165, mRNA	1868	98.95%	1698	XM_032859477.1
PREDICTED: *Lutra lutra* arcadia ring finger ubiquitin ligase 2C, mRNA	1862	98.85%	1891	XM_047698597.1
PREDICTED: *Meles meles* ring finger protein 165, mRNA	1862	98.85%	1654	XM_046025353.1
PREDICTED: *Enhydra lutris kenyoni* ring finger protein 165, mRNA	1851	98.66%	1660	XM_022495954.1
PREDICTED: *Halicho erus grypus* ring finger protein 165, mRNA	1829	98.28%	5449	XM_036107596.1

**Table 3:** Comparisonof the *RNF165* gene sequence of the individuals tested with the reference sequence from the Ensembl database.

Description	Max Score	Identity [%]	Length [bp]
6_Ex.1 ^2^	1923	99.90	1044
5_Ex.1 ^2^	1923	99.90	1044
3_Ex.1 ^1^	1923	99.90	1044
4_Ex.1 ^2^	1917	99.81	1044
2_Ex.1 ^1^	1917	99.81	1044
1_Ex.1 ^1^	1917	99.81	1044

^1^ Variant From the Wild Population. ^2^ Variant From the Farmed Population.

## Data Availability

All data are included in the manuscript; however, if further information regarding data availability is needed, the corresponding author will provide it upon special request.

## Supplementary Materials

The following supporting information can be downloaded at: https://www.mdpi.com/article/10.3390/genes16121417/s1, Figure S1: Chromatogram of exon 5 of the RNF165 gene obtained with the R primer in the first breeding group individual; Figure S2: Chromatogram of exon 5 of the RNF165 gene obtained with the F primer in the first breeding group individual; Figure S3: Alignment of RNF165 amino acid sequences in individuals 1–3 (breeding group) and 4–6 (wild group) relative to the reference sequence (0 Ex.1–8). Polymorphic site is marked with a red arrow; conserved sites with an asterisk.

## Abbreviations

The following abbreviations are used in this manuscript:

AMDV	Aleutian mink disease virus
AMD	Aleutian mink disease
CIEP	counterimmunoelectrophoresis
QTLs	quantitative trait loci
RNF165	RING Finger Protein 165
