# Supplementary figures and images for: Polymorphism of the RNF165 Gene in American Mink (Neogale vison) as a Potential Factor Responsible for Resistance to Infection with the Aleutian Mink Disease Virus
Source: Genes (Basel). 2025 Nov 28;16(12):1417. doi: 10.3390/genes16121417 (PMC12732447; doi:10.3390/genes16121417)

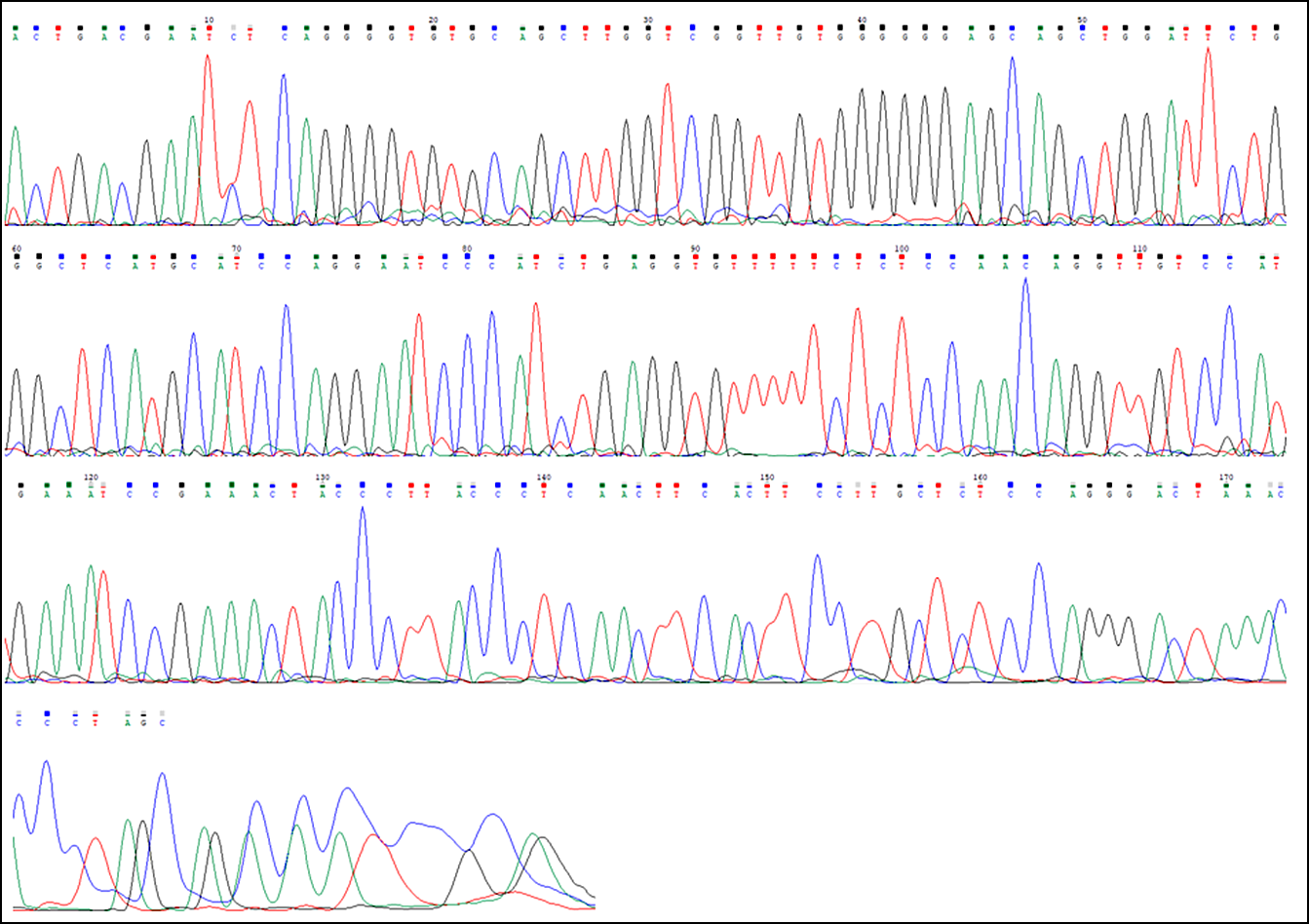

Supplement: Supplementary file 1 [file genes-16-01417-s001.zip › Figure S2.bmp]

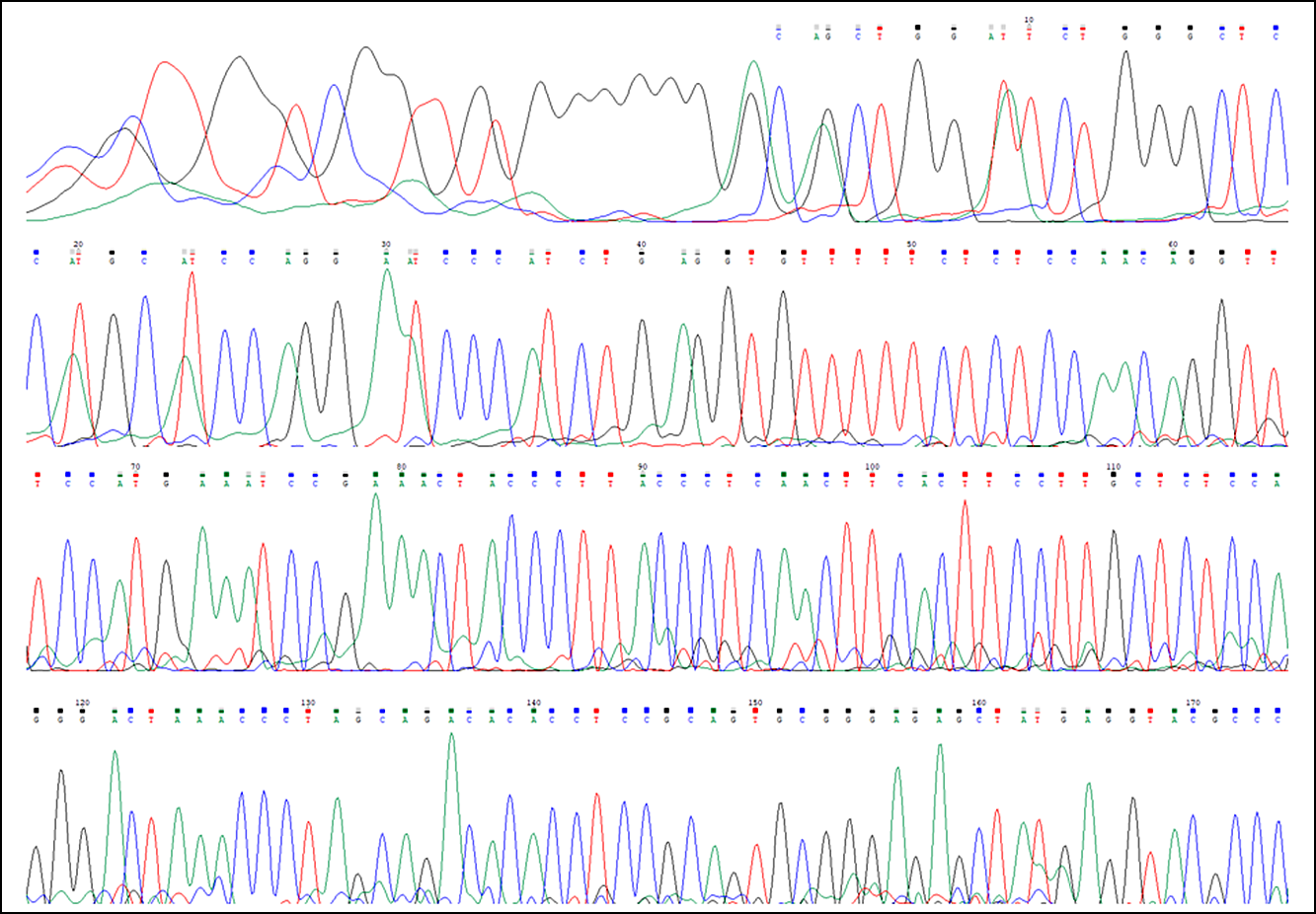

Supplement: Supplementary file 1 [file genes-16-01417-s001.zip › Figure S1.bmp]

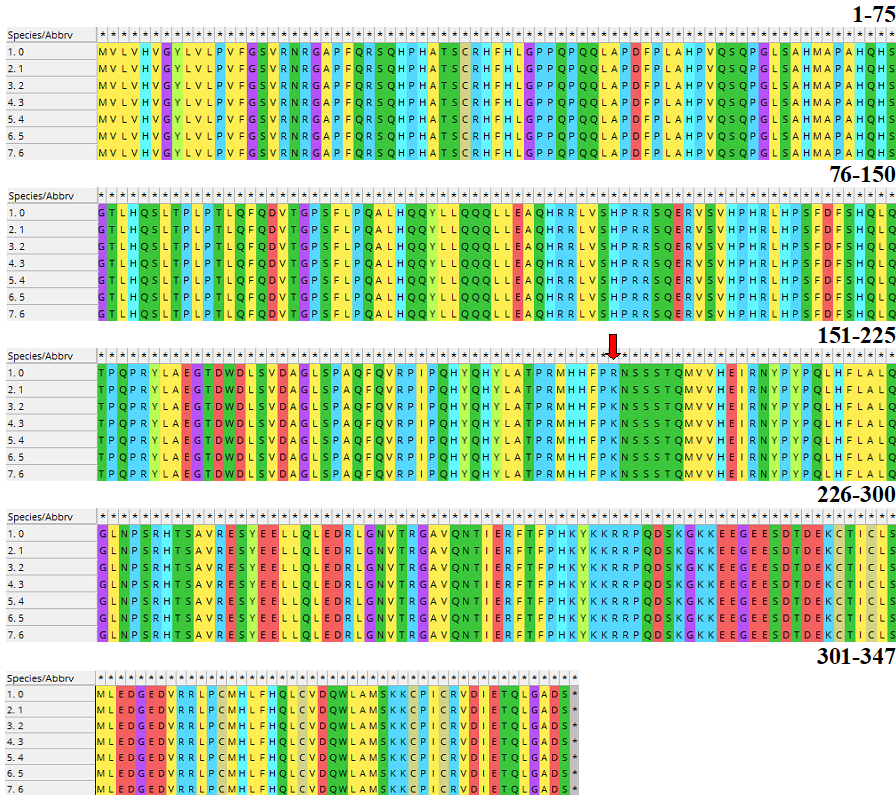

Supplement: Supplementary file 1 [file genes-16-01417-s001.zip › Figure S3.bmp]
